# New Approach to Hyponatremia: High Prevalence of Cerebral/Renal Salt Wasting, Identification of Natriuretic Protein That Causes Salt Wasting

**DOI:** 10.3390/jcm11247445

**Published:** 2022-12-15

**Authors:** John K. Maesaka, Louis J. Imbriano, Candace Grant, Nobuyuki Miyawaki

**Affiliations:** Department of Medicine and Division of Nephrology and Hypertension, NYU Langone Hospital Long Island, NYU Long Island School of Medicine, 200 Old Country Road, Suite 370, Mineola, NY 11501, USA

**Keywords:** cerebral/renal salt wasting, natriuretic peptide, hyponatremia, fractional urate excretion

## Abstract

Our understanding of hyponatremic conditions has undergone major alterations. There is a tendency to treat all patients with hyponatremia because of common subtle symptoms that include unsteady gait that lead to increased falls and bone fractures and can progress to mental confusion, irritability, seizures, coma and even death. We describe a new approach that is superior to the ineffectual volume approach. Determination of fractional excretion (FE) of urate has simplified the diagnosis of a reset osmostat, Addison’s disease, edematous causes such as congestive heart failure, cirrhosis and nephrosis, volume depletion from extrarenal salt losses with normal renal tubular function and the difficult task of differentiating the syndrome of inappropriate secretion of antidiuretic hormone (SIADH) from cerebral/renal salt wasting (C/RSW). SIADH and C/RSW have identical clinical and laboratory parameters but have diametrically opposite therapeutic goals of water-restricting water-loaded patients with SIADH or administering salt water to dehydrated patients with C/RSW. In a study of nonedematous patients with hyponatremia, we utilized FEurate and response to isotonic saline infusions to differentiate SIADH from C/RSW. Twenty-four (38%) of 62 hyponatremic patients had C/RSW with 21 having no clinical evidence of cerebral disease to support our important proposal to change cerebral to renal salt wasting (RSW). Seventeen (27%) had SIADH and 19 (31%) had a reset osmostat. One each from hydrochlorothiazide and Addison’s disease. We demonstrated natriuretic activity in the plasma of patients with neurosurgical and Alzheimer diseases (AD) in rat clearance studies and have now identified the natriuretic protein to be haptoglobin related protein without signal peptide (HPRWSP). We introduce a new syndrome of RSW in AD that needs further confirmation. Future studies intend to develop HPRWSP as a biomarker to simplify the diagnosis of RSW in hyponatremic and normonatremic patients and explore other clinical applications that can improve clinical outcomes.

## 1. Introduction

Hyponatremia, defined as a serum sodium of <135 mmol/L, is the most common electrolyte problem encountered in clinical practices worldwide and is an independent risk factor for higher morbidity and mortality rates [1,2]. Symptoms related to hyponatremia have been traditionally associated with acute reductions in serum sodium but there is awareness that even mild, chronic hyponatremia is associated with mental dysfunction, unsteady gait, osteoporosis, and 5-fold increases in falls and bone fractures, especially in the elderly [3,4,5,6]. Based on this awareness, most if not all hyponatremic patients are being treated. Selecting the appropriate treatment, however, depends on our ability to identify accurately the causes of hyponatremia that have diverse and even disparate treatment strategies. Our pathophysiologic approach to evaluating patients with hyponatremia has improved our ability to identify the different causes of hyponatremia and generated significant diagnostic insights that warrant an updated review of this evolving transformation.

## 2. It Is Time to Abandon the Outmoded Volume Approach

The diagnostic approach to hyponatremia has traditionally started with an assessment of the state of extracellular volume, designated as being euvolemic, hypervolemic or hypovolemic, despite a general acceptance that we cannot accurately assess the volume status of patients by usual clinical criteria [7].

Foremost among the limitations is to resolve the conundrum of differentiating “euvolemic” patients with the syndrome of inappropriate secretion of antidiuretic hormone (SIADH) from hypovolemic patients with cerebral/renal salt wasting (C/RSW) by this ineffectual and technically inaccurate volume approach, as all credible volume determinations in SIADH have shown them to be hypervolemic and not euvolemic [8,9,10,11]. Moreover, this diagnostic dilemma is compounded by having identical clinical and laboratory characteristics that are shared by both SIADH and C/RSW, Table 1. Both present with normal renal, adrenal and thyroid function, hyponatremia, hypouricemia, concentrated urine with urine sodium concentration (UNa) usually >30 mmol/L and increased fractional excretion (FE) of urate >11%, Table 1. Studies which utilize these criteria to confirm the diagnosis of SIADH without going through the rigors of differentiating SIADH from C/RSW, should, therefore, be met with legitimate skepticism. It might, however, be an impractical effort to resolve this diagnostic dilemma when C/RSW is considered to be a very rare or non-existent syndrome [12,13,14]. As discussed in previous publications, the perception of the rarity or non-existence of C/RSW is understandable because the original paper on salt wasting failed to prove such a syndrome existed [15,16]. It would be a wasted effort to go through the difficult process of differentiating SIADH from C/RSW if C/RSW is indeed a rare or non-existent syndrome. This diagnostic dilemma, however, needs to be resolved because we developed a more pathophysiologic approach over the past 30 years and found C/RSW to occur in 38% of hyponatremic patients in the general medical wards of the hospital [17]. The high prevalence of C/RSW, thus, creates an important therapeutic dilemma of deciding whether to water-restrict or administer the inhibitor of the V2 pitressin receptor to water-loaded hypervolemic patients with SIADH or administer salt and water to volume-depleted patients with C/RSW [17]. Application of this new pathophysiologic approach has improved identification of the different causes of hyponatremia with greater clarity. Moreover, the recent identification of haptoglobin related protein without signal peptide (HPRWSP) as the probable cause of C/RSW can serve as a biomarker to simplify the diagnosis of C/RSW on first encounter when initiation of saline infusions to these dehydrated patients will improve clinical outcomes [18].

## 3. Pathophysiologic Approach to Evaluate Hyponatremic Patients

### 3.1. Determination of FEurate

#### 3.1.1. Increased FEurate > 11%

**Figure 1:**
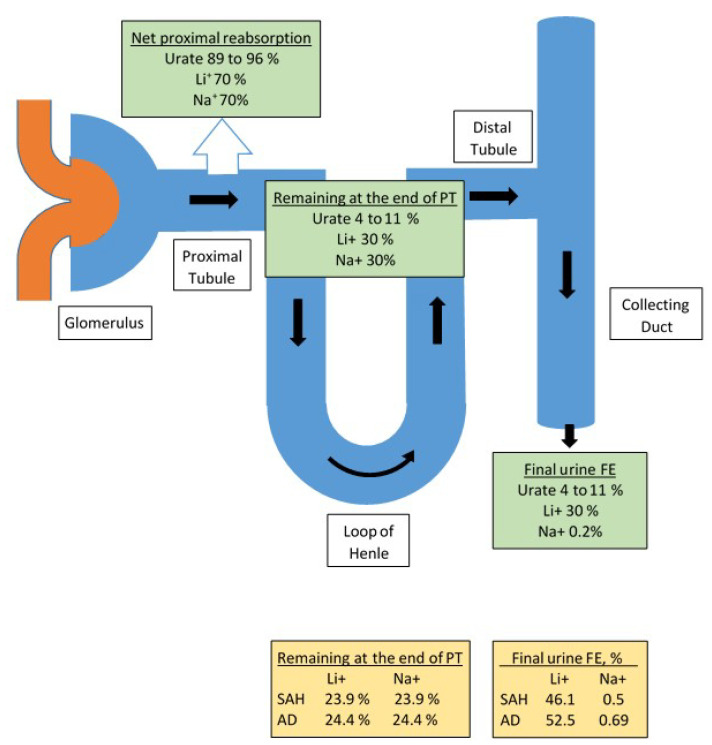
Figure depicting the handling of filtered urate, lithium and sodium by different segments of the renal tubule under normal (green) conditions and after infusion of serum from patients with RSW (yellow): one due to a subarachnoid hemorrhage and another due to Alzheimer’s disease. Net proximal reabsorption represents the percent reabsorption of urate, lithium (Li^+^) and sodium (Na^+^) in the proximal tubule. Remaining at the end of proximal tubule (PT) represents the percentage of each solutes filtrate entering the early Loop of Henle. Final urine FE represents the net handling of solute expressed as the fraction of filtered solute excreted in the urine. Yellow boxes indicate the percent of the filtered urate, lithium and sodium remaining at the end of the proximal tubule and in the final urine when rats were injected with serum of a patient with a subarachnoid hemorrhage (SAH) and another with Alzheimer’s disease (AD). Note the identical amount of sodium and lithium leaving the proximal tubule and how sodium is much lower than lithium in the final urine because sodium is reabsorbed by the distal tubule but not lithium.

Beck proposed that the coexistence of hyponatremia and hypouricemia, defined as serum urate < 4 mg/dL, differentiated SIADH from most other causes of hyponatremia [19]. As noted in Figure 1, urate is freely filtered at the glomerulus, is transported exclusively in the proximal tubule by reabsorptive and secretory transporters where the net reabsorption is 89 to 96% of the filtered urate with 4–11% remaining in the proximal tubule [20]. Since urate is not transported in the distal nephron, the 4–11% of the filtered urate load will be excreted in the final urine, referred to as FEurate. FEurate can be readily calculated by collecting blood and urine at the same time and utilizing the following formulae:

(1) $$\begin{array}{ll} FEurate\;in\;\% & =\frac{urine\;urate}{serum\;urate}÷\frac{urine\;creatinine}{serum\;creatinine}\times 100\\ & =\frac{urine\;urate\times serum\;creatinine}{urine\;creatinine\times serum\;urate}\times 100 \end{array}$$

**Figure 2:**
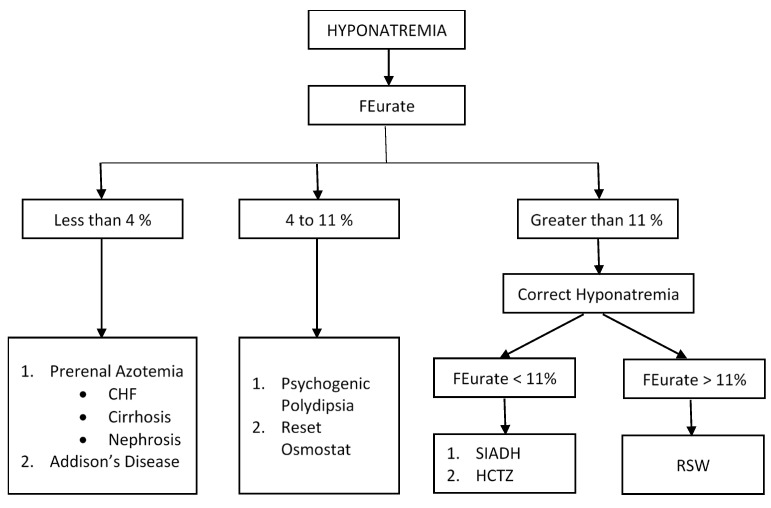
Algorithm utilizing FEurate to identify the different causes of hyponatremia. FEurate can be used to identify different causes of hyponatremia. Note the increased FEurate in patients with SIADH and RSW who have identical clinical and laboratory parameters when hyponatremic. The response to isotonic saline infusions in patients with increased FEurate >11% can be used to differentiate SIADH from RSW. Note how the changes in FEurate after correction of their hyponatremia with isotonic saline infusions can differentiate SIADH from RSW. See Figure 3.

Interestingly numerous studies have shown that the increased FEurate >11% in SIADH occurred only when the patient was hyponatremic and normalized to <11% after correction of the hyponatremia, Figure 2 [17,19,21,22,23,24]. We utilized this relationship between serum sodium and FEurate to differentiate SIADH from C/RSW by demonstrating a persistently increased FEurate after correction of the hyponatremia in C/RSW, Figure 2 [10,17,24,25,26]. Hydrochlorothiazide is known to increase FEurate probably in the same manner as in SIADH but with the basic understanding of the mechanisms contributing to drug induced hyponatremia as reviewed by Kim in this Special Issue, the role of FEurate in drug-induced hyponatremia remains to be defined [27].

#### 3.1.2. Normal FEurate of 4–11%

A reset osmostat (RO), which occurs in about 30% of hyponatremic patients, can be readily identified by having a normal FEurate of 4–11% [17,28,29]. RO is rarely identified as a cause of hyponatremia. It is usually diagnosed by demonstrating an occasional dilute urine in a spontaneously excreted urine or by a rarely performed water-loading test [28]. As noted in Figure 2, a normal FEurate has also been reported in patients with psychogenic polydipsia, which can be readily differentiated from RO because of the history of drinking large volumes of water and excreting large volumes of dilute urines [30]. We have now accumulated convincing data to conclude that a normal FEurate can readily identify patients with RO [17,28]. Treatment of RO is identical to SIADH.

#### 3.1.3. Decreased FEurate < 4%

Since urate is transported exclusively in the proximal tubule, the excretion rates of urate and other solutes will be low as seen in prerenal azotemic conditions where there is under perfusion of the kidneys and an increase in solute reabsorption in the proximal tubule [31]. In brief, prerenal azotemia is found in conditions where there is decreased perfusion of the kidneys and is characterized by an increase in solute reabsorption in the proximal tubule, excretion of concentrated urines, urine osmolality > plasma osmolality, low UNa < 20 mmol/L, increased BUN to creatinine ratio and increased plasma ADH levels [31]. As will be discussed later, the volume stimulus to ADH secretion is more potent than the osmolar stimulus, so a patient with hyponatremic prerenal azotemia will remain hyponatremic as long as they have decreased perfusion of the kidneys and are drinking water [32]. Prerenal azotemia is found in edematous states such as congestive heart failure (CHF), cirrhosis and nephrosis, in volume depleted states due to non-renal losses of solute such as vomiting and diarrhea with normal renal tubular function and in Addison’s disease, Figure 2.

The early diagnosis of Addison’s disease is not only important but very difficult to achieve because of nondescript signs and symptoms such as fatigue, weight loss, nausea, vomiting, postural dizziness and hyperpigmentation. As much as 50% are diagnosed when they present with life threatening adrenal crisis [33]. Of the routine laboratory tests performed, as much as 84% of patients present with serum sodium <137 mmol/L with only 34% presenting with potassium >5.0 mmol/L [34]. When presenting with hyponatremia, they usually present with normal BUN and creatinine, concentrated urine with UNa >30 mmol/L; so they are often misdiagnosed as SIADH, which can be devastating when these volume-depleted patients are water-restricted. In Addison’s disease, the mineralocorticoid deficiency will reduce sodium reabsorption in the distal tubule to reduce extracellular volume, which stimulates ADH secretion and induce hyponatremia, but are volume depleted in the presence of intact proximal tubule function. The proximal tubule will thus respond to the volume depletion by increasing solute reabsorption to induce a prerenal state with increased urate reabsorption and decreased FEurate of <4%. According to the algorithm in Figure 2 the low FEurate of <4% will lead to the diagnosis and treatment of Addison’s disease before they progress to the stage of adrenal crisis [24]. Because hyponatremia is much more common than hyperkalemia in Addison’s disease, the incorporation of determining FEurate in hyponatremic conditions will identify Addison’s disease at an earlier stage of the disease.

### 3.2. Response to Isotonic Infusions

The plasma levels of ADH are inappropriately increased in SIADH because ADH does not respond to the usual volume or osmolar stimuli. ADH in C/RSW, however, responds appropriately to both stimuli. This important physiologic difference can be appreciated by the dissimilar responses to isotonic saline infusions in SIADH as compared to C/RSW. As Bartter stated in 1967, “a striking and consistent finding in patients with SIADH is the persistence of hyponatremia even when large quantities of sodium are administered” [35]. Isotonic saline infusions have never been advocated to treat or correct the hyponatremia in SIADH. In contrast, patients with C/RSW have predictably consistent responses to isotonic saline infusions because of the appropriateness of the increase in plasma ADH levels. The response to isotonic saline can, thus, be predicted by applying the physiologic principle that the volume stimulus is more potent than the osmolar stimulus, Figure 4 [32]. So a volume depleted patient will have increased levels of ADH and remain hypo-osmolar or hyponatremic as long as they are hypovolemic and are drinking water, Figure 4. The infusion of isotonic saline will eliminate the more potent volume stimulus and allow the coexisting hypo-osmolality to inhibit ADH secretion, induce excretion of dilute urines and correct the hyponatremia. This phenomenon was utilized by Chung et al., who stated that an increase in serum sodium of >5 mmol/L during infusion of isotonic saline to a hyponatremic patient is consistent with hypovolemic hyponatremia [7]. This feature of the greater potency of the volume stimulus over the osmolar stimulus has not been clarified or systematically demonstrated.

**Figure 3:**
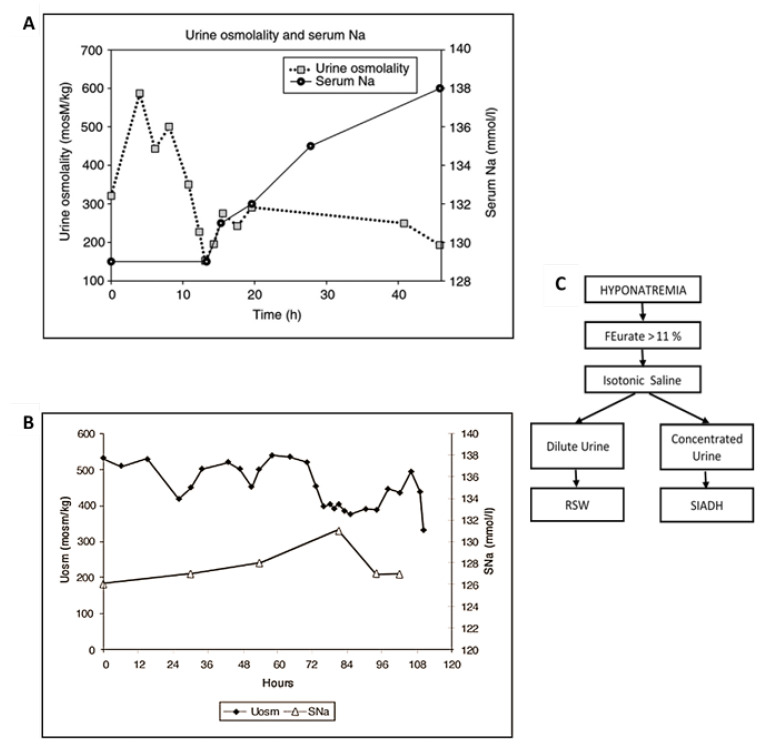
Effect of isotonic saline on urine osmolality. (**A**) The effect of isotonic saline infusion on urine osmolality and serum sodium in a volume depleted hip fracture patient without cerebral disease who had increased plasma levels of renin, aldosterone and ADH. Graph. Note progressive dilution of urine with undetectable level of ADH when urine was most dilute. Because water was removed from the body by excretion of dilute urines, serum sodium normalized within 48 h. (**B**) Graph showing response to isotonic saline infusion in a patient with SIADH who had increased blood volume and decreased plasma renin and aldosterone levels. Note the absence of dilute urines or correction of hyponatremia. This patient failed to inhibit ADH in the absence of volume depletion and presence of hypo-osmolality to meet criteria for SIADH. (**C**) Algorithm using urine osmolality response to isotonic saline to differentiate SIADH from RSW.

**Figure 4:**
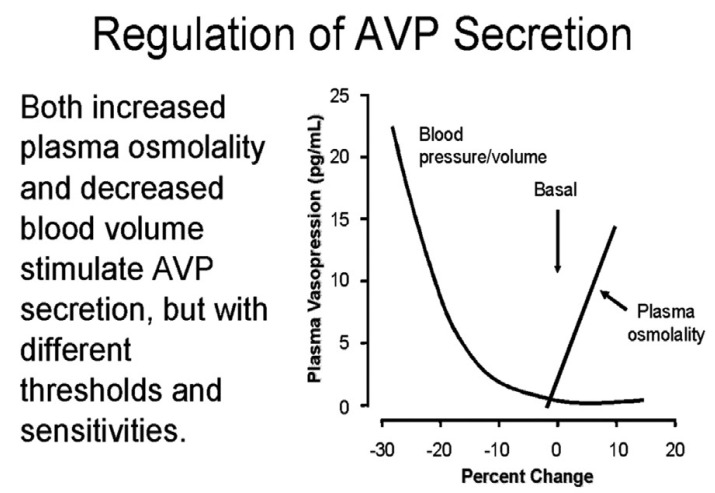
Graph demonstrating the progressive increase in ADH levels with worsening volume depletion despite the coexistence of plasma hypo−osmolality; the volume stimulus is more potent than the osmolar stimulus. This is why a volume depleted patient remains hyponatremic while drinking water. Isotonic saline infusions will eliminate the volume stimulus and allow the hypo−osmolality or hyponatremia to inhibit ADH secretion, get rid of the excess water and correct the hyponatremia.

Because of the difficulty in differentiating SIADH from C/RS, we decided to test the response to isotonic saline in unequivocally diagnosed cases of SIADH and C/RSW. We performed blood volume studies by radioisotope dilution methods utilizing 51 chromium labeled red blood cells and radio-iodinated serum albumin. Two patients with SIADH had increased blood volume, low plasma renin and aldosterone levels as compared to decreased blood volume and high plasma renin and aldosterone levels in C/RSW, which along with clinical and laboratory findings collectively strengthened the diagnoses of SIADH and C/RSW, respectively. Isotonic saline infusions in the 2 patients with SIADH failed to induce excretion of dilute urines or correct the hyponatremia as compared to diluting the urine after 13 h of isotonic saline infusions and correcting the hyponatremia within 48 h in the patient with C/RSW, Figure 3a,b [9,26]. These studies are consistent with previous reports that patients with SIADH do not dilute their urine or correct their hyponatremia by isotonic saline infusions as compared to diluting their urine and correcting the hyponatremia in C/RSW.

## 4. Utilization of FEurate and Response to Isotonic Saline in Evaluation of Hyponatremic Patients

We decided to determine whether hyponatremic patients with increased FEurate >11% diluted their urine and corrected their hyponatremia with infusions of isotonic saline and determined FEurate before and after correction of their hyponatremia in 62 hyponatremic patients recruited from the general medical wards of the hospital [17]. According to the algorithm presented in Figure 2, 17 (27%) had SIADH based on normalization of a previously increased FEurate after correcting their hyponatremia in 5 and 17 failing to dilute their urine or correct their hyponatremia after receiving ample volumes of isotonic saline. Twenty four (38%) had C/RSW. Twelve had persistently increased FEurate after correcting their hyponatremia, 19 had isotonic saline-induced dilute urines with 10 requiring infusions of D5W to prevent too rapid correction of their hyponatremia and osmotic demyelination syndrome [36]. Six of the 19 patients who had isotonic saline-induced dilute urines also had persistently increased FEurate after correction of their hyponatremia. Twenty one of the 24 patients did not have clinical evidence of cerebral disease to support our proposal to change cerebral to renal salt wasting (RSW) [37]. This change in nomenclature is extremely important because many C/RSW patients would otherwise not consider renal salt wasting in the absence of cerebral disease. We will thus use the more appropriate term, RSW, instead of C/RSW for the rest of this manuscript. Textbooks and many review articles on hyponatremia consider CSW, or more appropriately RSW, to be a rare clinical entity, so the majority of these patients are being fluid-restricted for an erroneous diagnosis of SIADH. This mistreatment of patients with RSW could in part account for the increased morbidity and mortality associated with hyponatremia. Nineteen (31%) had RO based on a normal FEurate in all patients and 8 had confirmatory spontaneously excreted dilute urines. It is interesting to note that baseline UNa <20 mmol/L was found in 10 patients with RSW as compared to 5 with a reset osmostat and 2 with SIADH, suggesting that patients with RSW had lost their appetite and consumed less sodium, probably because of the severity of their comorbid conditions. These patients would not have been considered to have RSW or SIADH by utilizing algorithms which state that UNa is >30 or >40 mmol/L [38,39]. Determining UNa is, therefore, less reliable when evaluating patients with hyponatremia. One each had hyponatremia due to hydrochlorothiazide and Addison’s disease [17].

It should be pointed out that it is extremely difficult to differentiate SIADH from RSW by the methods described above. FEurates must be determined after correction of the hyponatremia or isotonic saline infusions initiated with frequent urine osmolality determinations to see if isotonic saline infusions diluted or failed to dilute the urine when there are length of stay concerns. Successful execution of this difficult protocol was possible by a funded study, which provided the necessary personnel to perform these studies on a timely basis, especially the unexpected high prevalence of RSW in the general medical wards of the hospital. Challenges continue to confront us as we attempt to simplify methods to identify the different causes of hyponatremia, especially to differentiate RSW from SIADH.

## 5. Pathophysiology of RSW

RSW of the type described in this manuscript does not include other causes of salt wasting such as chronic kidney disease, Addison’s disease, Bartter’s or Gitelman’s syndromes. It occurs in patients with normal kidney function with creatinine not exceeding 1.3 mg/dL and normal thyroid function. It goes through two important physiologic phases that start with the upregulation of a natriuretic protein such as the recently identified HPRWSP by a multitude of cerebral and many non-cerebral conditions with or without hyponatremia [18].

### 5.1. Initiation of Nonequilibrated Phase

The natriuretic factor, HPRWSP, has its major effect on proximal tubule sodium transport to increase sodium and water excretion, which exceeds sodium and water intake to decrease extracellular volume. The extent of the volume depletion depends on the balance between the potency of the natriuretic factor in increasing salt and water excretion and salt and water intake. The deficits in sodium and water can be significant enough to cause hemodynamic instability with lower blood pressure and even postural hypotension with reflex tachycardia. This non equilibrated state is difficult to document in the majority of cases because of its short duration. This phase was documented in a 74 year old female hypertensive who sustained a subarachnoid hemorrhage. Her admission systolic blood pressure of 166 mmHg decreased to 98 and 93 mmHg when saline infusions lagged behind an increasing urine output between days 2 and 5. She received four 250 mL IV boluses of isotonic saline to maintain hemodynamic stability untilthe saline infusion matched the urine output of 4–5 L/day [18]. She was not hyponatremic at any time because infusion of isotonic saline has become a standard of care for SAH after reports of fluid restriction increasing morbidity and mortality [40]. The volume depletion appropriately increases the production of renin, aldosterone and ADH and reducing blood pressure and glomerular filtration rates as part of the activating humoral, hemodynamic and neural factors that attempt to reduce sodium and water excretion.

### 5.2. Equilibrated State

The patients eventually escape the effects of the salt wasting natriuretic protein by undergoing humoral, hemodynamic and neuronal compensations and enter an equilibrated state where sodium and water intake matches sodium and water output but at lower total body salt and water volumes. In this volume-depleted state sodium excretion and even serum sodium concentration will depend on the intake of sodium and water. As noted in our 62 hyponatremic patients, 10 of the 24 patients with RSW had UNa <20 mmol/L at a time when they were in the equilibrated phase when sodium input equals output and in a state where their comorbid conditions were severe enough to reduce appetite and sodium intake [17]. An unappreciated aspect of hyponatremic conditions is the need to have sufficient water intake to induce hyponatremia. This is examplified by the inability to induce hyponatremia in healthy humans and dogs given daily injections of pitressin without increasing water intake [41,42]. Since humans lose approximately 500 mL of pure water as insensible water losses each day, we will all become hypernatremic without sufficient water intake. Hyponatremia can only occur when water intake exceeds insensible water losses. Infusions of isotonic saline appears to correct the volume depletion partially as a large part of the infused saline will significantly increase urine output along with very troublesome nocturia. Fortunately, the duration of RSW appears to be of short duration as the comorbid condition subsides. The most effective treatment is to develop an inhibitor to the natriuretic protein, HPRWSP, which would eliminate the distressing urinary frequency and nocturia.

## 6. Identification of Haptoglobin Related Protein without Signal Peptide as Natriuretic Factor in RSW

We previously injected the plasma of 21 patients with neurosurgical diseases and 18 AD patients into rats and demonstrated significant increases in FENa, FElithium and urine flow rates without changing blood pressure or glomerular filtration rates as compared to normal and gender-matched controls in both studies and vascular dementia in the AD study [43,44]. Because urate is exclusively transported in the proximal tubule, we decided to investigate lithium transport because lithium is transported mainly in the proximal tubule on a one to one basis with sodium, Figure 1 [45]. LIthium can thus be a reliable marker of sodium transport in the proximal tubule. Significant advancements in protein analysis over the ensuing 25 years allowed us to identify the natriuretic protein that was present in the serum of a salt wasting patient due to a subarachnoid hemorrhage (SAH) and another due to AD. We accomplished this by performing rat renal clearance studies after injecting the rats with natriuretic activity in their sera [18]. In these studies, FENa increased from 0.2 to 0.5 and 0.69% and FELi increased from 23.9 to 46.1% and 24.4 to 52.2% in the SAH and AD patients, respectively [18]. Since lithium is transported one to one in the proximal tubule with sodium, the 46.1 and 52.2% FELi estimates the amount of sodium being delivered to the distal tubule. The FENa of 0.5 and 0.69% in the final urine suggests that 45.6 of the 46.1 and 51.51 of the 52.2% of the filtered sodium was reabsorbed by the distal nephron in the SAH and AD studies, respectively [18]. These data suggest that the natriuretic protein has a profound effect on proximal tubular sodium transport as well as demonstrating the robust capacity of the distal nephron to transport sodium.

**Figure 5:**
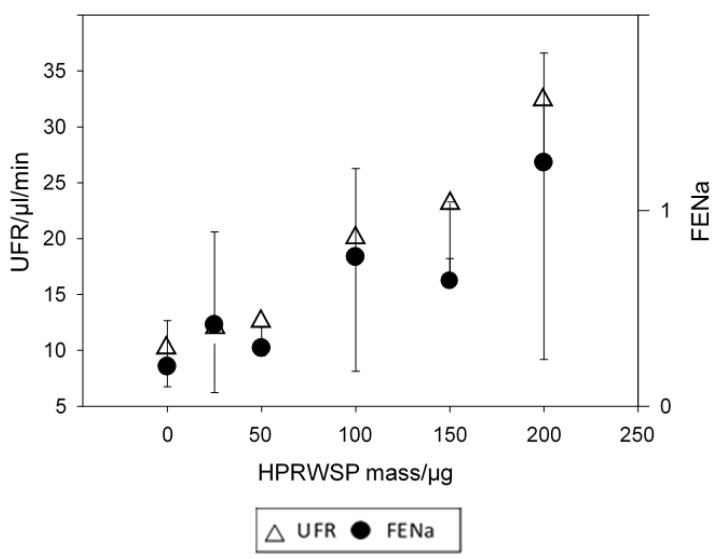
Graph showing the effect of increasing dose of the natriuretic protein, haptoglobin related protein without signal peptide on fractional excretion of sodium (FENa) and urine flow rates (UFR). Note the robust increase in both with increasing doses of the natriuretic protein.

**Figure 6:**
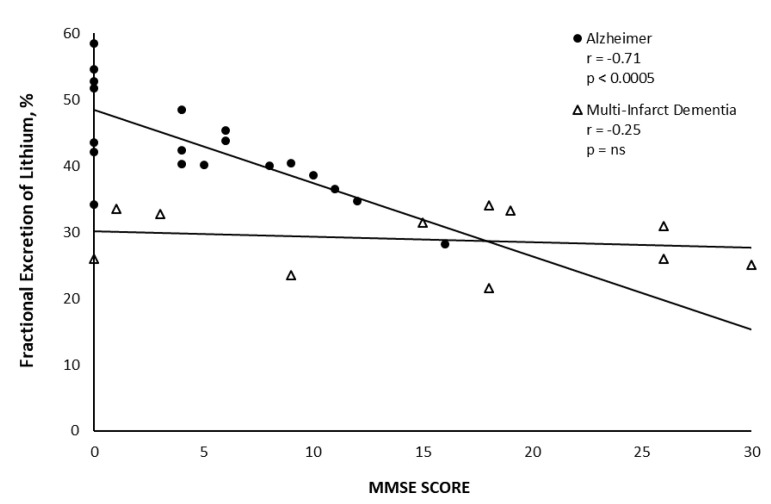
Graph showing the relationship between fractional excretion of lithium (FElithium) and mini mental state examination (MMSE) scores in patients with Alzheimer’s disease and multi−infarct dementia. Note the increasing FElithium as MMSE is decreasing. Because there is a dose response to the natriuretic factor, blood levels of the natriuretic factor either increased or the patient became more dehydrated as the dementia worsened.

Utilization of the sophisticated protein analysis revealed levels of haptoglobin related protein to be highest as compared to control sera. Haptoglobin related protein with signal peptide did not have natriuretic activity but HPRWSP was the only protein with natriuretic activity in SAH and AD sera. As noted in Figure 5, HPRWSP had a dose response with significant increases in FENa and urine flow rate [18]. Because FENa and urine flow rates increased as the dose of HPRWSP was increased, the progressive increase in FElithium with worsening dementia in AD suggests that blood levels of HPRWSP were also increasing progressively, Figure 6 [44]. These data suggest that all AD patients with mini-mental state examination (MMSE) of less than 12 are not only dehydrated but become progressively more dehydrated as their dementia worsens. These data suggest that a new syndrome of RSW not only exists in moderately demented patients with AD but also worsens as they become more demented. Future studies must estimate the existence and prevalence of RSW at different stages of their dementia and determine whether HPRWSP can serve as a biomarker to identify patients with RSW. It will be interesting to determine the effect of inhibiting HPRWSP on mental and physical function in AD.

It is our hope that these data will be an impetus for others to study the effect of this relatively unknown protein in many organs in the body, including brain.

## 7. RSW Occurring without Hyponatremia, Especially Alzheimer’s Disease and Subarachnoid Hemorrhage

Blood volume studies in patients with subarachnoid hemorrhage revealed 8 of 9 hyponatremic and 8 of 12 normonatremic patients to be volume depleted, suggesting that RSW occurs commonly in hyponatremic and normonatremic patients with SAH [11]. Water restricting hyponatremic patients with SAH has been shown to increase morbidity and mortality by increasing vascular spasm to increase ischemia and infarction of the brain. [40] It is now standard practice to administer isotonic saline to these and possibly other patients with neurosurgical diseases to reduce the likelihood of developing hyponatremia. RSW is probably present in many normonatremic patients.

Hyponatremia in AD is also an unusual occurrence because these patients limit their water intake by the natural reduction in thirst with aging in addition to the dementia that reduces water intake for multiple reasons. As stated above, RSW is not only present but appears to worsen as the MMSE levels decrease below 12 and future studies must determine the prevalence of RSW at all stages of dementia and whether HPRWSP can serve as a biomarker of RSW in AD. It should be noted that the identification of HPRWSP as the natriuretic factor in RSW was accomplished in normonatremic SAH and AD patients. The essential development of HPRWSP as a biomarker for RSW will give us the opportunity to identify RSW in hyponatremic and a potentially large group of normonatremic patients.

HPRWSP is the first legitimate inhibitor of proximal tubular sodium transport and can significantly mobilize fluid overload in patients with congestive heart failure when combined with a distal diuretic such as furosemide. Congestive heart failure induces pre renal azotemia by increasing the reabsorption of sodium and other solutes in the proximal tubule, which reduces the effectiveness of distal diuretics. As noted, the significant increases in FElithium quantitates the profound effect on proximal tubular sodium transport so its combination with the traditional distal diuretics will significantly increase sodium and water excretion with removal of the excess fluid in heart failure. The benefit of combining acetazolamide, a weak proximal diuretic, with furosemide in patients with acute heart failure improved outcomes and decreased length of stay [46]. Combining HPRWSP with a distal diuretic in patients with acute decompensated CHF and even CHF in an outpatient setting has the potential of improving outcome in CHF.

## 8. Conclusions

The development of a physiologically derived approach to hyponatremia has brought clarity in identifying many causes of hyponatremia with consequential improvement in clinical outcomes as we apply the appropriate management for each group of patients. The physiology of these conditions have many subtle nuances that must be addressed by well-founded physiologic principles that lead to credible outcomes. As previously discussed, the dictum that conclusions are based on data would be better expressed as conclusions being based on credible data [47]. We feel we have fortified our conclusions by presenting credible data that can withstand opposing views that have little credible support [13,14,47]. Unfortunately, the outcome of our research requires application of physiologic principles that are labor intensive and difficult to achieve, especially determining FEurate when the patient is hyponatremic and normonatremic or determining whether infusions of isotonic saline dilute the urine or not and whether it significantly increases serum sodium concentration. When we accomplished the physiologically derived new approach, however, the unexpected outcome was to uncover a very high prevalence of RSW that was considered rare or nonexistent in hyponatremic patients in the general medical wards of the hospital. These well designed and executed studies uncovered many nuances in hyponatremic and normonatremic patients to lead to the following credible conclusions.

1. RSW is common in the general medical wards of the hospital
2. Change cerebral salt wasting to RSW. Twenty one of the 24 patients with RSW did not have clinical evidence of cerebral disease. RSW would not be considered without the presence of cerebral disease. We must incorporate this important change in nomenclature.
3. RSW can occur in hyponatremic and in a potentially large number of normonatremic patients.
4. We have introduced and support the conclusion that RSW is not only a new syndrome in AD but is probably common.
5. Determining urine sodium concentrations in work up of hyponatremia is not as informative as professed to be. It should be viewed as being less informative.
6. The identification of HPRWSP as the natriuretic protein that probably causes RSW can have the following clinical applications:
  a. Serve as a biomarker of RSW to simplify the diagnosis of RSW in a wide variety of comorbidities, including a new syndrome of RSW in AD, on first encounter with the patients and to deliver the appropriate management and improve clinical outcomes.
  b. Because increasing salt and water intake in patients with RSW will increase excretion of large urine volumes that includes a distressing nocturia, there is a need to develop an inhibitor to HPRWSP to improve outcomes.
  c. HPRWSP satisfies our long search for a potent proximal diuretic, which can be combined with a distal diuretic to effectively eliminate the fluid overload of congestive heart failure and improve clinical outcomes.

## Figures and Tables

**Table 1:** Clinical and laboratory findings common to SIADH and RSW.

Association with intracranial diseases Hyponatremia Concentrated urine Urinary [Na] usually >30 mEq/L Normal renal/adrenal/thyroid function Non-edematous Hypouricemia, Increased fractional excretion of urate Only difference is volume status

Table listing identical clinical and laboratory findings in SIADH except for their volume status. All parameters are used clinically to characterize both diseases. One key difference that can-not be assessed clinically is the volume status, being increased in SIADH and decreased in RSW.

## Data Availability

No new data were created or analyzed in this review. Data sharing is not applicable to this article.
